# Primary Hydatid Cyst of the Pancreas: A Literature Review on a Rare and Challenging Occurrence of Echinococcosis

**DOI:** 10.7759/cureus.60797

**Published:** 2024-05-21

**Authors:** Alin Mihetiu, Dan Georgian Bratu, Dan Sabau, Hassan Noor, Alexandra Sandu

**Affiliations:** 1 Second Surgical Department, Faculty of Medicine, "Lucian Blaga" University of Sibiu, Emergency County Hospital of Sibiu, Sibiu, ROU; 2 Surgery, Faculty of Medicine, "Lucian Blaga" University of Sibiu, Sibiu, ROU; 3 General Surgery, Faculty of Medicine, "Lucian Blaga" University of Sibiu, Sibiu, ROU

**Keywords:** pancreatic tail, echinococcosis, scolicidal agents, obstructive jaundice, open surgery, primary pancreatic hydatid cyst, zoonosis

## Abstract

Hydatid disease is caused by the *Echinococcus* tapeworm and is a zoonosis that endemically affects certain geographic areas with a high prevalence in animal husbandry. Due to globalization, the pathology can also be encountered beyond these preferred geographic areas. It predominantly affects the liver and lungs, with pancreatic localizations of hydatid cysts being rare and posing a challenge for differential diagnosis and surgical tactics. The present study aimed to provide a recent scoping of the literature on this type of localization, analyzing demographic data, therapeutic management, and postoperative outcomes. It was observed that females are more frequently affected in pancreatic hydatid localizations (p < 0.001), with the most common symptomatology represented by abdominal pain. The preferred localization was at the level of the pancreatic tail (32.5%), followed by cephalic localizations (25%). The preferred surgical approach was open surgery, with an observed preference for open surgery in specific localizations, such as the head, isthmus, and body of the pancreas (p < 0.001). Radical procedures are more commonly used than conservative ones (52.5% vs. 47.5%), and paradoxically, although less invasive, procedures such as inactivation and drainage are associated with more frequent complications (p = 0.03). This type of localization, due to the elements of local anatomical topography, requires adequate preparation in biliopancreatic surgery, considering that sometimes preoperative diagnosis is not oriented, and intraoperative records may require extensive interventions. Our research encompassed a thorough review of literature spanning the last decade using PubMed and Google Scholar databases, focusing specifically on cases involving primary hydatid cysts found within the pancreas. Thirty-three relevant articles were published between 2014 and 2024. In addition, we presented a unique case study that illustrates this uncommon occurrence.

## Introduction and background

Hydatid disease represents an endemic zoonosis, with its highest incidence distributed in geographic areas where animal husbandry is extensively conducted under non-industrialized conditions. The most common etiological agents of this condition are *Echinococcus granulosus* and *Echinococcus multilocularis*. Within the parasite's life cycle, humans serve as intermediate hosts, with anatomical involvement primarily manifesting in the liver (70-75%), lungs (20%), spleen (0.5%-8%), and kidneys (2%-4%) [1,2]. Hydatid localization within the pancreas is a rarely encountered scenario, particularly in its primary form. The incidence of this localization ranges between 0.14% and 2% in endemic areas and remains less than 1% in reported cases of hydatidosis [3,4].

Distribution of pancreatic hydatid cysts is more frequent in the head of the pancreas (50%), followed by the body (24-34%), and less commonly in the tail (16-19%). Notably, around 90% of pancreatic hydatid cysts present as solitary lesions [5].

The etiopathogenesis of this localization of *Echinococcus* infection is controversial, with several theories being postulated. Hematogenous dissemination is the most accepted pathogenic mechanism. Other mechanisms suggest that the parasite traverses through absorption from the intestinal lumen into pancreatic veins or is implanted through passage from the bile duct into the pancreatic duct at the bile-pancreatic confluence [6,7,8].

Clinically, the manifestation of this condition is often nonspecific, with cases frequently discovered incidentally or presenting as acute pancreatitis, obstructive jaundice, or obstructive symptoms due to compression of the adjacent digestive tract segments. Epigastric pain, accompanied by nausea or vomiting, typically constitutes the primary symptomatology.

The course is protracted, and cyst development in the omental bursa can lead to various complications, primarily through compressive mechanisms on the pancreatic duct, intrapancreatic bile duct, neighboring vessels (superior mesenteric, splenic), or adjacent organs - stomach, duodenum, and colon. Another less frequent complication is the rupture of the cyst into the biliary tract, pancreatic duct, or peritoneal cavity [1,8].

**Figure 1:**
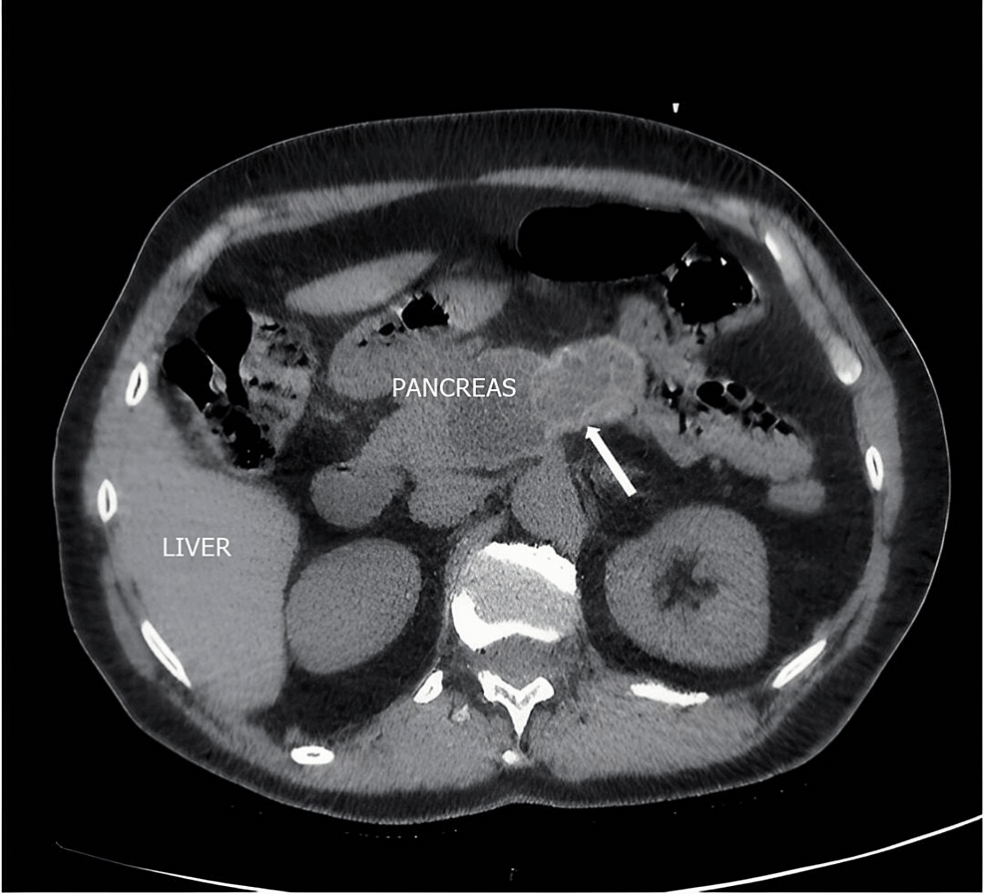
Pancreatic hydatid cyst (indicated by a white arrow): CT image in the axial section (personal case study).

**Figure 2:**
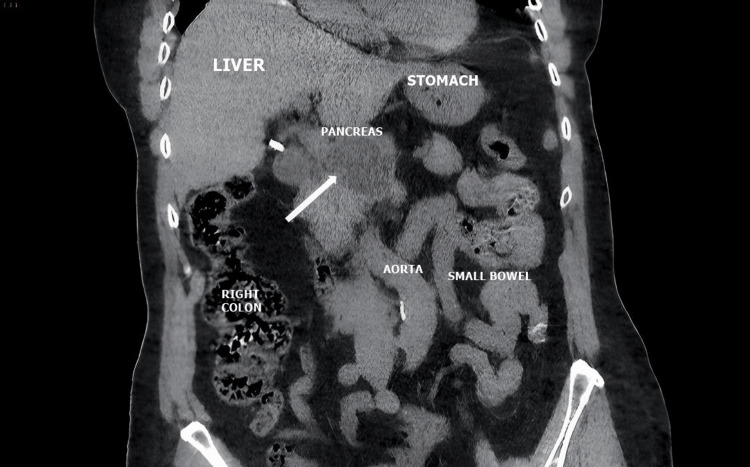
Pancreatic hydatid cyst (indicated by the white arrow): CT images in the coronal section (personal case study).

**Figure 3:**
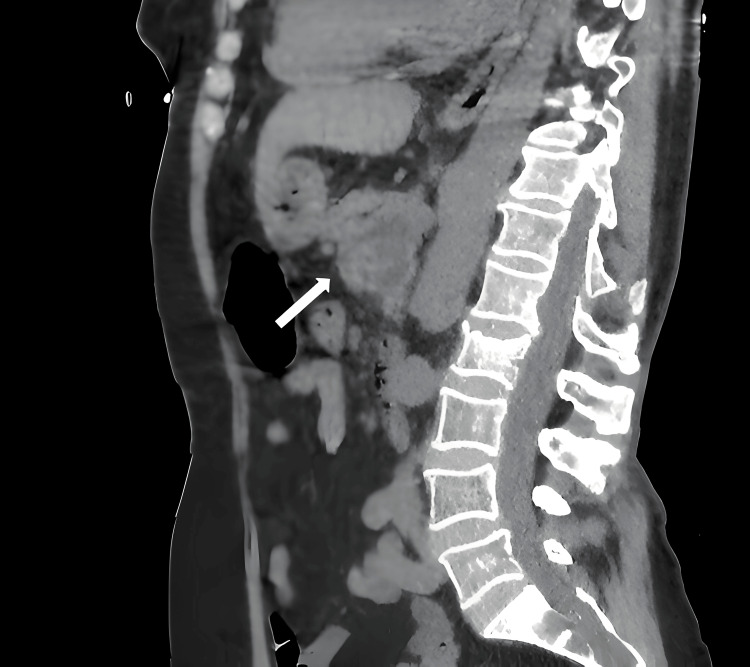
Pancreatic hydatid cyst (indicated by the white arrow): CT image in the sagittal section (personal case study).

Imaging with CT scan or MRI can pose challenges in differential diagnosis, as pancreatic hydatid disease is often confused with pancreatic pseudocysts or pancreatic cystadenomas and much less commonly with pancreatic cystadenocarcinomas (Figures 1, 2, 3) [9].

The percutaneous approach using PAIR (Puncture, Aspiration, Injection, and Reaspiration) represents a rarity due to the risk of intraprocedural contamination, inadequate cyst evacuation, or complications arising from proximity to important vascular structures [10].

Another potential approach involves the endoscopic transgastric method for cysts protruding into the posterior gastric wall. However, despite being theoretically plausible, this approach lacks substantial supporting evidence, especially in comparison to its established use in pancreatic pseudocyst management.

Pharmacological treatment serves primarily as a preoperative measure or as prophylaxis against postoperative recurrence. Singular pharmacological therapy for this particular localization lacks sufficient evidence to substantiate its efficacy in achieving complete healing.

The study aimed to conduct a comprehensive review of the literature concerning pancreatic hydatid localization, focusing on demographic data, therapeutic approaches, and postoperative results. It specifically examined the prevalence among females, typical symptoms, and preferred surgical methods.

## Review

We conducted a comprehensive literature review covering the past decade, focusing on cases of primary hydatid cysts in the pancreas. In addition, we presented a case study illustrating this rare occurrence, where initial imaging suggested a pancreatic pseudocyst, but intraoperative findings revealed its hydatid origin.

By searching the PubMed and Google Scholar databases using the keywords "primary hydatid cyst” and “pancreas," we identified a total of 33 relevant articles published between 2014 and 2024, comprising 15 from PubMed and 18 from Google Scholar. The inclusion criteria encompassed case reports or case series delineating this specific localization, as well as original research articles. We considered adult cases from documents containing demographic data (age and sex), localization specifics, symptomatology, imaging investigations regarding cyst localization and size, surgical details (including approach type and technique), and postoperative outcomes. Exclusion criteria encompassed non-English cases, pediatric cases, extra-pancreatic or peripancreatic localizations not originating from the pancreatic parenchyma, and articles lacking accessible or complete data.

Moreover, cases involving multiple pancreatic localizations or the coexistence of hepatic hydatid cysts with pancreatic ones were excluded due to the inability to definitively establish their primary origin. To minimize the risk of bias concerning the selected study data and the interpretation of surgically presented outcomes, two authors independently analyzed the results. The agreement ratio was 100% for the included articles and 98% for those excluded. Given the limited case reports in the specialized literature, extensive original research studies or meta-analyses were not accessible. Therefore, we focused on case reports and case series.

We gathered and processed the data using IBM SPSS Statistics for Windows, version 28.1 (released 2021, IBM Corp., Armonk, NY), along with the DATAtab Team's online statistics calculator (2024). T-tests, Chi-square tests, Pearson correlation tests, and linear regression analysis were employed to compare various demographic, preoperative, operative, and postoperative data. Results with a p-value <0.05 were interpreted as statistically significant. These tools played a crucial role in enabling us to derive evidence-based conclusions.

Approval was obtained from the Committee on Research Ethics of the University Lucian Blaga of Sibiu as it was conducted in accordance with the Declaration of Helsinki for studies involving humans. Written informed consent has been obtained from the patients to publish intraoperative aspects. 

**Figure 4:**
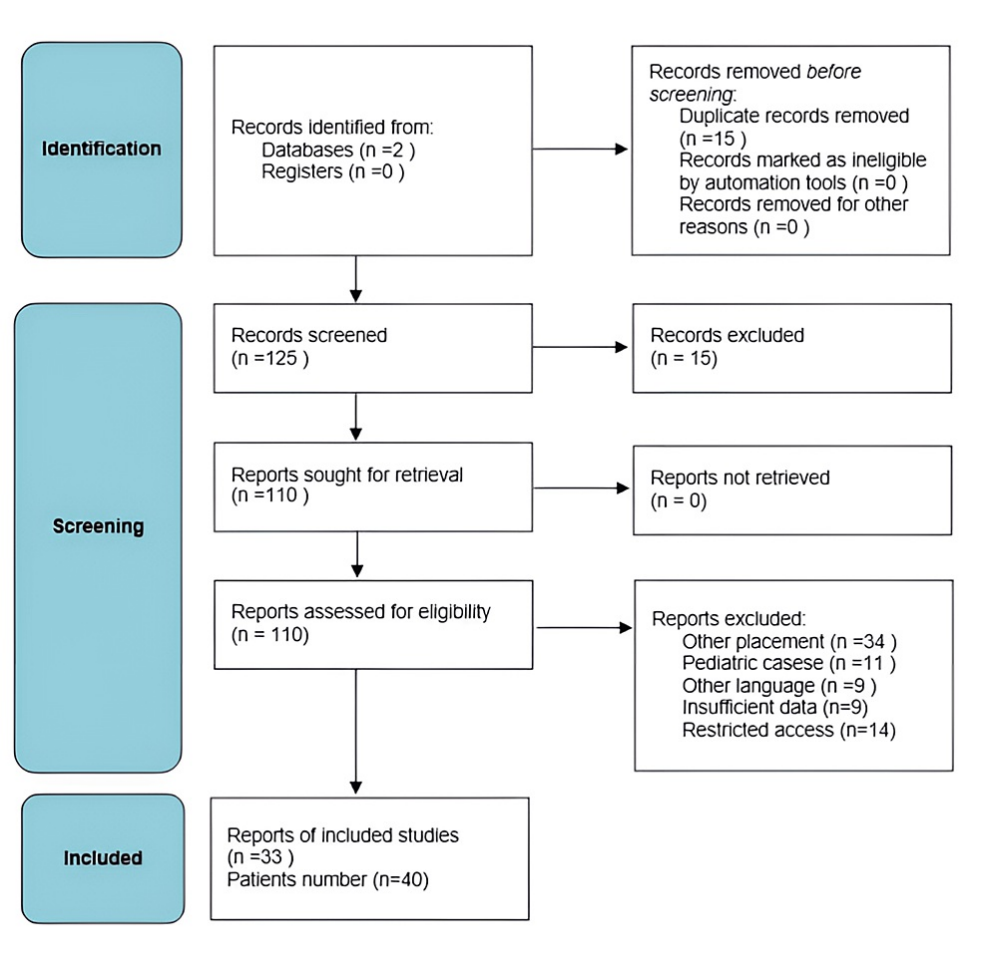
PRISMA (Preferred Reporting Items for Systematic Reviews and Meta-Analyses) flow chart on the literature review regarding pancreatic hydatid cyst.

Few literature review studies address this specific localization of hydatid cysts, and most provide only a synthesis with basic statistical data and lack exhaustive statistical analysis. The most recent reports of this nature date back a decade, necessitating a review of additional cases, especially regarding advancements in imaging and minimally invasive surgical treatment. Not all reviewed articles provide reports on the type of scolicidal agent used or the duration of hospitalization. While the impact of the former parameter on recurrence or complications may be limited, reporting hospitalization duration would have been beneficial, especially for comparing open surgery to laparoscopy. This review adhered to the PRISMA (Preferred Reporting Items for Systematic Reviews and Meta-Analyses) guidelines (Figure 4, Table 1).

**Table 1:** Pancreatic hydatid cyst literature scoping results. CT: computed tomography, MRI: magnetic resonance imaging, ERCP: endoscopic retrograde cholangiopancreatography

Author	Patient number	Country	Sex	Age	Symptoms	Preoperative imaging	Placement	Size	Surgical approach	Inactivation solution	Surgical management	Postoperative complications
Vasilescu et al. [11]	1	Romania	F	64	Pain	CT	Body	11/8 cm	Laparoscopy	Serum hypertone	Inactivation, drainage	None
Ahmed Z et al. [4]	1	India	F	40	Pain	CT	Body	5/5 cm	Open surgery	0.5% Cetrimide	Inactivation, drainage	None
Kothiya PK et al. [12]	1	India	F	20	Pancreatitis	CT, MRI, ERCP	Head	7/6 cm	Laparoscopy	10% Povidone-iodine	Inactivation, deroofing, drainage	Pancreatic fistula
Cherouaqi Y et al. [13]	1	Morocco	F	54	Pain	MRI	Isthmus	5/3 cm	Laparotomy	-	Inactivation, drainage	None
Wu Y et al. [14]	1	China	F	28	Jaundice	CT,MRI,ERCP	Head	6/5 cm	Laparotomy	-	Cephalic duodenopancreatectomy	None
Hiremath B et al. [15]	1	India	F	-	Epigastric mass	CT	Neck	10/8 cm	Laparotomy	-	Inactivation, partial cystectomy, drainage	Pancreatic fistula
Deák J et al. [16]	1	Hungary	M	34	Pain	CT	Head	13 cm	Laparotomy	-	Cystectomy	None
Kısaoğlu A et al. [17]	1	Turkey	F	58	Pain	MRI	Body and tail	5/3 cm	Laparotomy	-	Splenectomy, distal pancreatectomy	None
Qarmo MM et al. [3]	1	Syria	M	33	Pain	CT	Head	11/3 cm	Laparotomy	Hypertonic saline	Inactivation, partial cystectomy, drainage	None
Alsaid B et al. [18]	1	Syria	M	34	Pancreatitis	CT	Body	13/4 cm	Laparotomy	-	Inactivation, deroofing, drainage	Retroperitoneal abscess, acute pancreatitis
Sethi S et al. [19]	1	India	F	48	Pain	CT	Tail	14/13 cm	Laparotomy	-	Inactivation, drainage	None
Jajal V et al. [2]	1	India	F	32	Pain	CT/ERCP	Head	4/5 cm	Laparoscopy	Hypertonic saline	Inactivation, drainage	None
Elaffand A et al. [20]	1	Egypt	M	34	Epigastric mass	MRI	Body and tail	11/8 cm	Laparotomy	Hypertonic saline	Cystogastrostomy	None
Attash SM et al. [21]	1	Iraq	F	22	Pain	CT	Body and tail	15/7 cm	Laparotomy	-	Cystogastrostomy	None
Akbulut S et al. [22]	1	Turkey	F	47	Nausea	CT	Body and tail	12/10 cm	Laparotomy	-	Splenectomy, distal pancreatectomy	None
Kumar R et al. [23]	1	India	F	49	Pain	CT	Tail	5/5 cm	Laparotomy	10% Povidone-iodine	Cystectomy	None
Ozsay O et al. [24]	1	Turkey	F	23	Pain	CT	Tail	5/4 cm	Laparotomy	-	Splenectomy, distal pancreatectomy	None
Hashemi Fard A [25]	1	Iran	F	55	Pain	CT	Tail	6/4 cm	Laparoscopy	0.5% Cetrimide	Inactivation, drainage	None
Makhoul E et al. [26]	1	Lebanon	F	52	Pain	CT	Body and tail	8/5 cm	Laparotomy	-	Distal pancreatectomy	None
Soin P et al. [27]	1	India	F	40	Pain	CT	Body	7/5 cm	Laparotomy	Hypertonic saline	Inactivation, drainage	None
Mattous M et al. [28]	1	Morocco	F	30	Pain	CT	Head	10/8 cm	Laparotomy	-	Inactivation, drainage	None
Mabrouk MB et al. [29]	1	Tunisia	F	25	Pain	CT	Tail	6 cm	Laparotomy	-	Cystectomy	None
	1	-	F	32	Epigastric mass	CT	Tail	15 cm	Laparotomy	-	Pericystectomy	None
	1	-	M	41	Pain	CT	Tail	15 cm	Laparotomy	-	Pericystectomy, dplenectomy	none
	1	-	M	29	Jaundice	CT	Head	7 cm	Laparotomy	-	Pericystectomy	None
	1	-	F	25	Pain	CT	Body and tail	9 cm	Laparotomy	-	Splenectomy, distal pancreatectomy	None
	1	-	F	42	Jaundice	CT	Head	6 cm	Laparotomy	-	Cystectomy	None
	1	-	F	24	Pain	CT	Body	8 cm	Laparotomy	-	Cystectomy	None
	1	-	F	35	Pain	CT	Body	5 cm	Laparotomy	-	Cystectomy	None
Hasnaoui A et al. [30]	1	Tunisia	F	73	Pain	CT	Tail	9 cm	Laparotomy-	-	Inactivation, drainage	None
Belhaj A et al. [31]	1	Morocco	F	19	Pain	CT/MRI	Body	-	Laparotomy	-	Medial pancreatectomy	Pancreatic fistula
Mitrovic M et al. [32]	1	Serbia	F	76	Pain	CT	Tail	11 cm	Laparoscopy	-	Distal pancreatectomy	None
Ansari Z et al. [33]	1	India	F	55	Pain	CT	Tail	8/7 cm	Laparotomy	-	Distal pancreatectomy	None
Lada PE et al. [5]	1	Argentine	F	18	Epigastric mass	CT	Head	12 cm	Laparotomy	-	Inactivation, drainage	None
Zarbaliyev E et al. [34]	1	Turkey	F	70	Pain	CT	Tail	7/5 cm	Laparotomy	-	Distal pancreatectomy	None
Destek S et al. [35]	1	Turkey	M	45	Pain	CT	Body and tail	9/8 cm	Laparoscopy	-	Splenectomy, distal pancreatectomy	None
Afilal I et al. [36]	1	Morocco	M	42	Epigastric mass	CT/MRI	-	15/6 cm	Laparotomy	-	Splenectomy, distal pancreatectomy	None
Patil R et al. [37]	1	India	F	45	Epigastric mass	CT	Head	9/5 cm	Laparotomy	-	Cephalic duodenopancreatectomy	Pancreatic fistula
Imam A et al. [38]	1	Israel	F	18	Pain	CT	Tail	4 cm	Laparoscopy	-	Distal pancreatectomy	None
Shrestha B et al. [39]	1	Nepal	F	51	Pain	CT	Body	5/4 cm	Laparotomy	-	Splenectomy, distal pancreatectomy	None

Among the 33 reports analyzed, a total of 40 patients were identified, with an average age of 39.92 ± 15.34 years. Pancreatic hydatid cysts were more commonly found in females (80%), a distribution that showed statistical significance (p < 0.001). The mean age was 40.78 ± 16.9 years for females and 36.5 ± 5.48 years for males.

The primary symptom observed in affected individuals was pain (70%), followed by the presence of an epigastric mass (15%), jaundice (7.5%), and 5% of patients displaying clinical and biological features suggestive of acute pancreatitis. In addition, 2.5% of patients reported nausea as the sole symptom.

Computed tomography (CT) was the predominant imaging modality used (70%), followed by magnetic resonance imaging (MRI) (7.5%) and a combination of CT and MRI. Hydatid cysts in the pancreas were most commonly located in the tail (32.5%), followed by the pancreatic head (25%). The distribution of hydatid cysts based on their localization within pancreatic segments, along with the sizes of the cystic formations, is detailed below (Table 2).

**Table 2:** Hydatid cyst distribution on pancreas segments.

Placement	Frequency	Percentage (%)	Mean	Minimum	Maximum	Mean ± Std.
Tail	13	32.5%	9.23	4	15	9.23 ± 4.25
Head	10	25%	8.5	4	13	8.5 ± 2.95
Body	8	20%	8	5	13	8 ± 3.07
Body and tail	7	17.5%	9.86	5	15	9.86 ± 3.18
Isthmus	2	5%	7.5	5	10	7.5 ± 3.54

**Figure 5:**
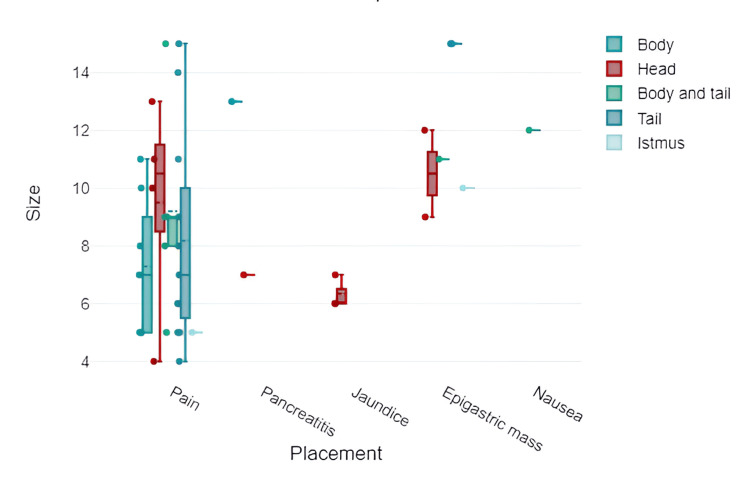
Graphic representation of the relation between the size, placement, and symptoms of pancreatic echinococcosis.

It is observed that the caudal and corporeal-caudal localizations developed the largest dimensions; however, no statistically significant changes or differences were detected (p > 0.05) (Figure 5).

Upon comparative analysis of symptoms based on localization, it was observed that pancreatic head localizations predispose more to pain (p = 0.021). In addition, cephalopancreatic localizations more frequently evolve with jaundice, pancreatitis, and palpable formations (p = 0.021).

In the majority of cases, surgical intervention was performed via open surgery (82.5%), through median laparotomy or subcostal incision, while the laparoscopic approach was utilized in 17.5% of patients.

A significant interdependence was observed between the type of surgical approach and the localization within pancreatic segments (p < 0.001), with laparotomy being preferred in 87.5% and 80% of cases with localization in the body and head of the pancreas, respectively, as well as in corporeal-caudal localizations (85.71%). Laparoscopy was most commonly used in tail of pancreas localizations (23.08%).

It was observed that radical procedures were utilized in 52.5% (n = 21) of cases, while conservative approaches were employed in 47.5% (n = 19) of patients. The average size of cysts approached conservatively was 9.96 cm, whereas for those approached radically, it was 7.92 cm. There was a tendency to opt for radical procedures in cases of corporeal-caudal hydatid localizations, while cephalic and isthmic localizations benefited from conservative approaches (Table 3).

**Table 3:** Surgical type treatment in relation to cyst placement.

Placement	Conservatory	Radical	Total
Body	50% (n = 4)	50% (n = 4)	8
Head	60% (n = 6)	40% (n = 4)	10
Body and tail	28.57% (n = 2)	71.43% (n = 5)	7
Tail	38.46% (n = 5)	61.54% (n = 7)	13
Isthmus	100% (n = 2)	0%	2
Total	19	21	40

The rationale behind this lies in the technical challenges posed by the unique anatomical features and the considerably more intricate reconstruction techniques required in cephalopancreatic and isthmic localizations. The predominant surgical technique utilized was splenectomy and distal pancreatectomy (17.5%), followed by inactivation and drainage (15%) (Table 4).

**Table 4:** Surgical technique for pancreatic hydatid cyst.

Surgical management	Body	Head	Body and tail	Tail	Isthmus
Inactivation, drainage	37.5%	30%	0%	23.08%	50%
Inactivation, deroofing, drainage	12.5%	10%	0%	0%	0%
Cephalic duodenopancreatectomy	0%	20%	0%	0%	0%
Cystectomy	25%	20%	0%	15.38%	0%
Splenectomy, distal pancreatectomy	12.5%	0%	57.14%	15.38%	0%
Inactivation, partial cystectomy, drainage	0%	10%	0%	0%	50%
Cystogastrostomy	0%	0%	28.57%	0%	0%
Distal pancreatectomy	0%	0%	14.29%	30.77%	0%
Pericystectomy	0%	10%	0%	7.69%	0%
Pericystectomy, splenectomy	0%	0%	0%	7.69%	0%
Medial pancreatectomy	12.5%	0%	0%	0%	0%

Statistically significant values were obtained for approaches involving distal pancreatectomy and splenectomy in corporeal-caudal and caudal localizations (p = 0.013).

Postoperative complications were detected in 12.5% of cases, represented by pancreatic fistulas (10%) and a retroperitoneal abscess (2.5%). Cranial, isthmic, or strictly corporeal localizations were associated with a higher number of postoperative complications compared to caudal ones (10% vs. 2.5%).

Although laparoscopic approaches are seemingly associated with a higher percentage of complications per procedure (14.29%) reported concerning the total number of surgical interventions, they recorded a lower complication rate compared to open approaches (2.5% vs. 7.5%).

Paradoxically, conservative procedures recorded a higher number of postoperative complications, 15.79% (n = 3), compared to radical ones (9.52%, n = 2), although without achieving statistical significance (p > 0.05).

Analyzing each technique comparatively with postoperative complications, a higher risk of pancreatic fistula development was observed in cases where inactivation, de-roofing, and drainage were practiced (p = 0.03).

Discussion

Pancreatic hydatid cyst represents a rare localization of *Echinococcus* infection, occurring in a complex area where vascular, biliary, and digestive elements intersect. Older literature data suggest a more frequent involvement of males and the pancreatic head. However, our analysis, along with more recent studies, indicates a significantly higher incidence among females and a predominantly caudal localization of the hydatid lesion [14].

The surgical approach to such a localization requires thorough knowledge of hepatobiliary and pancreatic surgery. Depending on the local topography of anatomical elements involved, surgical intervention can range from simple drainage to partial or total pancreatectomies.

The anatomical location of the pancreas and the challenging approach under ultrasound or CT guidance make percutaneous interventions like PAIR a rarity, with their use being feasible only in large cysts with wall expression rather than retropancreatic ones. Even in these cases, the risk of injury to the tubular digestive organs or spillage remains high, thus limiting the widespread adoption of this method [10].

The surgical treatment of this pathology follows the standardized principles of hepatic or abdominal hydatid surgery. This involves isolating the operative field, inactivating the cystic content with scolicidal agents, and aspirating the contents. The scolicidal agents used are the same as those in hepatic hydatid surgery and have maintained widespread use, including hypertonic saline and alcohol. Solutions such as Cemetride, Iodopovidone, and silver nitrate have fallen out of common use due to the increased risk of associated complications such as parenchymal necrosis or cholangitis.

Although there are no studies specifically addressing the behavior of these scolicidal substances in relation to the pancreas, it can be considered by extension that a similar approach to that of the liver is preferable [40]. The preference for hypertonic saline as the primary scolicidal agent is also reflected in our analysis (44.44%).

**Figure 6:**
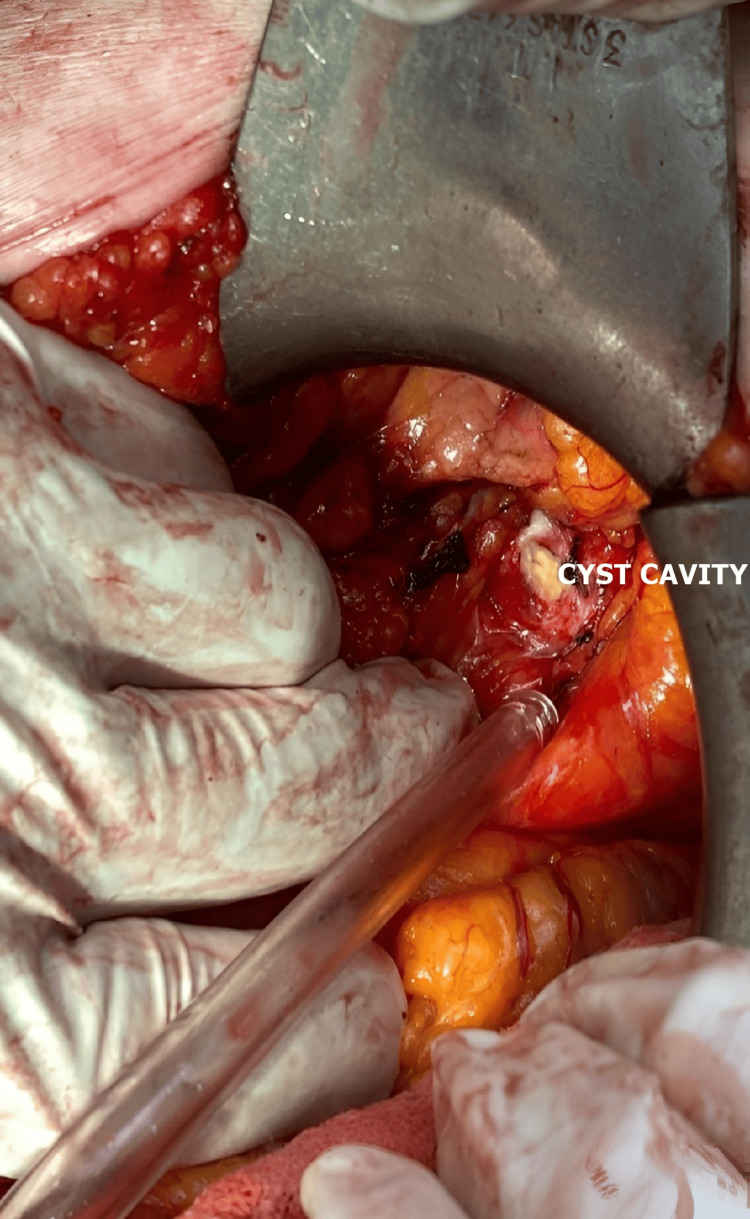
Intraoperative aspect of pancreatic hydatid cysts (personal case series).

**Figure 7:**
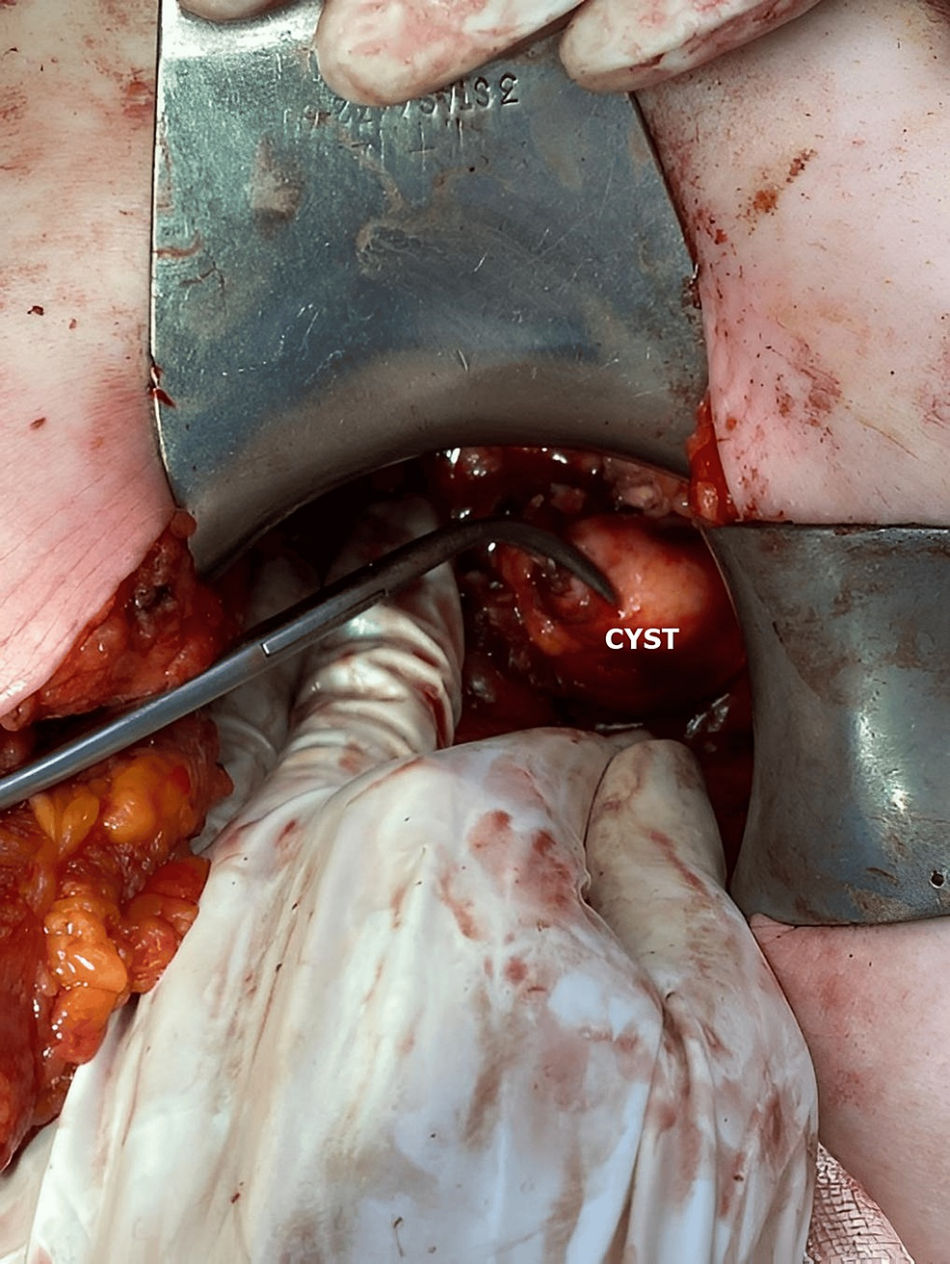
Intraoperative aspect of pancreatic hydatid cysts (personal case series).

**Figure 8:**
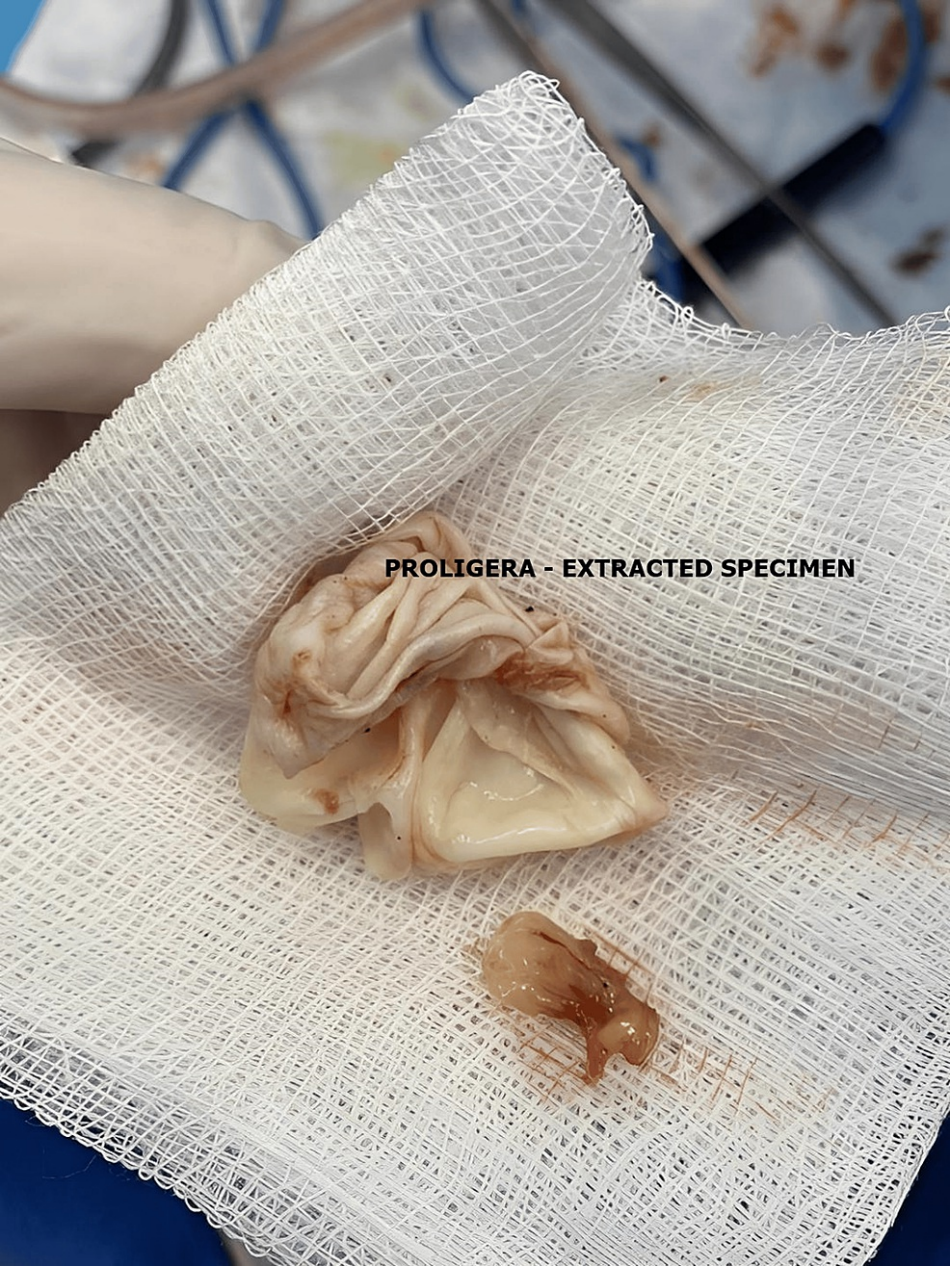
Intraoperative aspect of pancreatic hydatid cysts (personal case series).

Regarding the approach to the cyst, procedures can vary similarly to those for the liver. A conservative approach involves simple drainage, drainage with cystopericystectomy, or pericystectomy (Figures 6, 7, 8). Radical procedures aim for the complete removal of the cyst by performing cystectomy or partial organ resections, such as cephalic duodenopancreatectomy, caudal pancreatectomy with or without splenectomy, median corporal pancreatectomy, Frey or Beger pancreatectomy, or total pancreatectomy.

The present study highlights a slight tendency to opt for radical procedures over conservative ones. This inclination could be attributed to the identification of preoperative ambiguous imaging and the risk of conservatively treating a neoplastic cystic formation, such as pancreatic cystadenocarcinoma, mucinous or solid cystic neoplasm, or lesions with malignant potential, such as branch duct or main duct intraductal papillary mucinous neoplasms (IPMN) [14,41,42].

In such scenarios, radical intervention is justified. Otherwise, we believe that hydatid disease can be effectively managed with conservative surgery, sparing the patient from major organ or systemic interventions, which could increase mortality and morbidity.

Literature analysis has also revealed a preference for radical approaches in corporeal-caudal and cephalic localizations, justified by technically easier surgical interventions [43].

The laparoscopic approach to these hydatid localizations is less common (17.5% of cases) and more frequently employed in the tail of pancreas localizations. This approach offers significant benefits over open surgery, which typically involves extensive incisions, such as median or subcostal laparotomies, predisposing patients to wound infections, foreign body reactions such as seromas and granulomas, and incisional hernias [44].

However, the use of open surgery is justified when cyst localizations are challenging, there is a risk of spillage, other neighboring elements (especially vascular structures) are involved, or when the surgeon's experience in laparoscopic pancreatic surgery is limited [45,46].

Laparoscopic surgery in hydatid pathology presents fewer limitations compared to the percutaneous approach and is credited with superior outcomes over open surgery regarding operative duration, complications, hospitalization costs, and patient satisfaction. Initially restricted to hepatic localizations, laparoscopy is now indicated for extrahepatic localizations such as splenic, peritoneal, and pancreatic hydatid cysts [36,38,47,48].

A notable concern surrounding laparoscopic hydatid surgery is the risk of intraoperative contamination, although this can be effectively mitigated through the use of scolicidal-soaked materials or specialized instruments designed for hydatid procedures [49,50]. Postoperative complications of pancreatic hydatid cysts mirror those observed in hepatic hydatid disease, commonly involving pancreatic fistulas, hemorrhages, and abscesses.

A promising development in the realm of pancreatic hydatid cyst surgery is the increasing adoption of robotic-assisted procedures, offering a new dimension to the field of pancreatic surgery and hydatid disease management [51,52,53]. Diagnosing pancreatic hydatid cysts poses clinical and radiological challenges, with non-surgical treatment options offering limited efficacy. Surgical intervention necessitates careful consideration of cyst localization within the pancreatic segments, along with the associated risks of intraoperative and postoperative complications. Procedures aiming for radical resection or extensive interventions should be undertaken by experienced surgical teams.

## Conclusions

In summary, primary pancreatic involvement in hydatid disease is infrequent. Imaging diagnosis of this condition may sometimes prompt considerations of alternative types of pancreatic cystic formations, ranging from benign to malignant, thereby shaping surgical strategies accordingly. While open surgery remains the preferred surgical treatment for this localization, there is a growing trend toward minimally invasive surgical approaches, particularly with the increasing expertise in laparoscopic pancreatic surgery.

Further investigation may yield additional insights about the possibility of intraoperative contamination or may focus on investigating novel approaches intended to reduce postoperative complications.
